# Modeling the Bone Marrow Microenvironment to Better Understand the Pathogenesis, Progression, and Treatment of Hematological Cancers

**DOI:** 10.3390/cancers17152571

**Published:** 2025-08-04

**Authors:** Kathryn A. Skelding, Daniel L. Barry, Lisa F. Lincz

**Affiliations:** 1 Cancer Cell Biology Research Group, School of Biomedical Sciences and Pharmacy, College of Health, Medicine and Wellbeing, The University of Newcastle, Newcastle, NSW 2308, Australia; daniel.l.barry@uon.edu.au (D.L.B.); lisa.lincz@calvarymater.org.au (L.F.L.); 2 Precision Medicine Research Program, Hunter Medical Research Institute, Newcastle, NSW 2305, Australia; 3 Hunter Haematology Research Group, Calvary Mater Hospital Newcastle, Newcastle, NSW 2298, Australia

**Keywords:** bone marrow microenvironment, hematological cancers, hematological malignancies, co-culture, stromal cells, spheroids, bioreactors

## Abstract

Despite significant advancements in understanding the pathogenesis and treatment of hematological malignancies, including leukemia and multiple myeloma, the majority of patients continue to experience poor long-term outcomes. This is partly due to the difficulty of accurately recapitulating the malignant microenvironment in vitro, particularly the bone marrow niche. The complexity of the bone marrow microenvironment poses a challenge for the in vitro examination of hematological malignancies. Traditionally, 2D culture and animal models have been utilized, but these representations are limited and have been criticized for their lack of human physiological relevance. In an attempt to overcome this, 3D models have been developed that more accurately recapitulate the in vivo microenvironment. Herein, we present an overview of recent developments in 2D and 3D models used for studying the bone marrow niche in hematological malignancies, highlighting their advantages and limitations.

## Simple Summary

Relapse and treatment resistance remain a major concern for hematological malignancies. Despite treatment advancements, many patients still have poor long-term outcomes. This is partly because current preclinical models do not always accurately mimic the in vivo environment. Herein, we review recent advancements in using 3D in vitro models to study hematological malignancies, focusing on models that better reflect human physiological conditions. Improved models could lead to better-informed research and, ultimately, more effective treatment for patients.

## 1. Hematological Malignancies

Hematological malignancies encompass a broad group of diseases that arise due to defects in hematopoiesis, and account for approximately 6.5% of all cancers [1], including leukemias, lymphomas, multiple myeloma (MM), myelodysplastic syndromes (MDS) and myeloproliferative neoplasms (MPN). By 2030, the number of hematological malignancies is projected to reach approximately 4.6 million cases globally [2]. While the mortality rate for leukemias has decreased over the last 30 years, the mortality rates of other hematologic malignancies appear to have remained stable. To better improve mortality rates and patient outcomes, a better understanding of disease pathogenesis and response to treatment is required.

While preclinical hematological models have improved in recent decades, models that faithfully recapitulate the bone marrow microenvironment are still in their infancy. Improved models that mimic living orthotopic microenvironments will lead to a better understanding of hematological malignancy pathophysiology and drug response, and ultimately to improved patient outcomes.

## 2. The Bone Marrow Niche Is a Complex and Heterogeneous Organ

The bone marrow niche is a complex heterogeneous cellular support system within the marrow of the long and axial bones whose primary function is to support hematopoiesis. It is comprised of various hematopoietic and non-hematopoietic cells, including osteoblasts and osteoclasts, mesenchymal stromal cells (MSCs), neurons, immune cells, adipocytes, hematopoietic stem cells (HSCs), sinusoidal endothelium and perivascular stromal cells [3]. Additionally, structural components, including collagen fibers/fibrils and non-collagen molecules, act to build a meshwork in which these cellular components are embedded.

**Figure 1:**
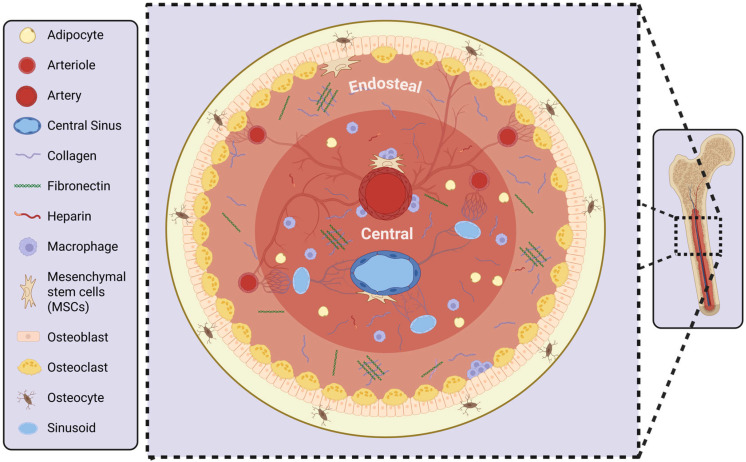
Schematic of the bone marrow niche. A representation of healthy bone marrow and its distinct niches: the Vascular niche—containing collagen, fibronectin, and mesenchymal stem cells (MSC), the Central niche—containing adipocytes, collagen, heparin, and macrophages, and the Endosteal niche—containing collagen, fibronectin, osteoclasts, and osteocytes. Generated using BioRender (https://BioRender.com/zn1w5tp (accessed on 31 July 2025)).

Anatomically, there are three distinct regions of the bone marrow niche: the vascular, central, and endosteal niches (Figure 1). The vascular/perisinusoidal niche promotes proliferation and differentiation, while the endosteal/osteoblastic niche supports quiescence and self-renewal. The cellular composition in each of these distinct regions is different [4], and hematopoietic cell subsets localize to distinct locations based on the stage of differentiation. During homeostasis, the HSCs are located close to the vasculature, whereas transplanted HSCs preferentially home to the endosteum [5]. Further, hematopoietic lineage commitment appears to be differentially regulated in these different niches [6]. This suggests that gradients of secreted factors or other events within the niche may contribute to the function of cells within the niche. These important chemical and physical factors require consideration when modeling the bone marrow.

Recent studies have demonstrated that the bone marrow is largely an extremely hypoxic environment. Different regions of the niche have different oxygen levels and gradients [4,7]. This hypoxic environment is determined by cellular levels and oxygen consumption rates in various areas of the niche. Chronic intermittent hypoxia can induce vascular remodeling in the bone marrow niche and modulate hematopoiesis [8]. The local oxygen tension within the bone marrow dramatically changes following radiation and chemotherapy [7], indicating that stress can alter the metabolic microenvironment, further highlighting the complexity of this niche.

As the major site of hematopoiesis, the bone marrow continuously generates a variety of hematopoietic cells and simultaneously maintains immune memory and harbors immune cells. The cellular composition of the niche is dynamic in nature and adapts to the requirements of the cells interacting within it. While stromal cells are the structural backbone of this niche, they also play central roles in immunomodulation, inflammation, and cancer [9]. Tumor-associated inflammation induces alterations in inflammatory signaling in the bone marrow niche, resulting in altered immune cell composition, and has also been implicated in the progression from MDS to acute myeloid leukemia (AML), different phenotypes and treatment responses in lymphoma, and disease progression in MM [10,11,12,13].

Cellular components within the niche modulate neighboring cells through direct contact and paracrine and immunomodulatory signaling [14,15]. However, during aging-related processes, the functions of cells, particularly MSCs, change, which leads to a senescent state [16]. This modulates niche factors, which, in turn, alter the functional state of cells within the bone marrow microenvironment. Importantly, within the hematopoietic system, senescent stromal cells have been implicated in the remodeling of the normal bone marrow niche into a pro-leukemia/myelodysplastic environment [16,17,18,19,20,21,22,23].

As cells develop the capability to leave the supportive bone marrow niche, key changes occur to increase their adhesive, migratory, and invasive potential. Despite developments in this area, these changes remain largely incompletely profiled. This is particularly true for extramedullary disease in MM, which remains challenging to study.

It is increasingly being recognized that perturbations in the bone marrow microenvironment can lead to the development and progression of hematological malignancies and myeloproliferative disorders, as well as mediate response to treatment [24,25,26,27,28]. However, despite this enhanced understanding, these findings have generally not translated into improved clinical outcomes. This is largely because the majority of preclinical models currently used to examine cancer cell biology and drug efficacy are highly simplistic: two-dimensional (2D) culture of homogenous immortalized cells grown as a single layer on plastic in a temperature-, oxygen-, and humidity-controlled atmosphere. Such commonly used cell lines demonstrate differential gene expression by up to 30% compared to their tissues of origin [29]. Although leukemia and lymphoma derived cell lines maintain better gene expression representation compared to solid tumors [30], these 2D culture models still lack the complexity of the natural cellular milieu, and do not even begin to mimic the intricacies of the bone marrow microenvironment.

## 3. Modeling the Bone Marrow Niche

**Figure 2:**
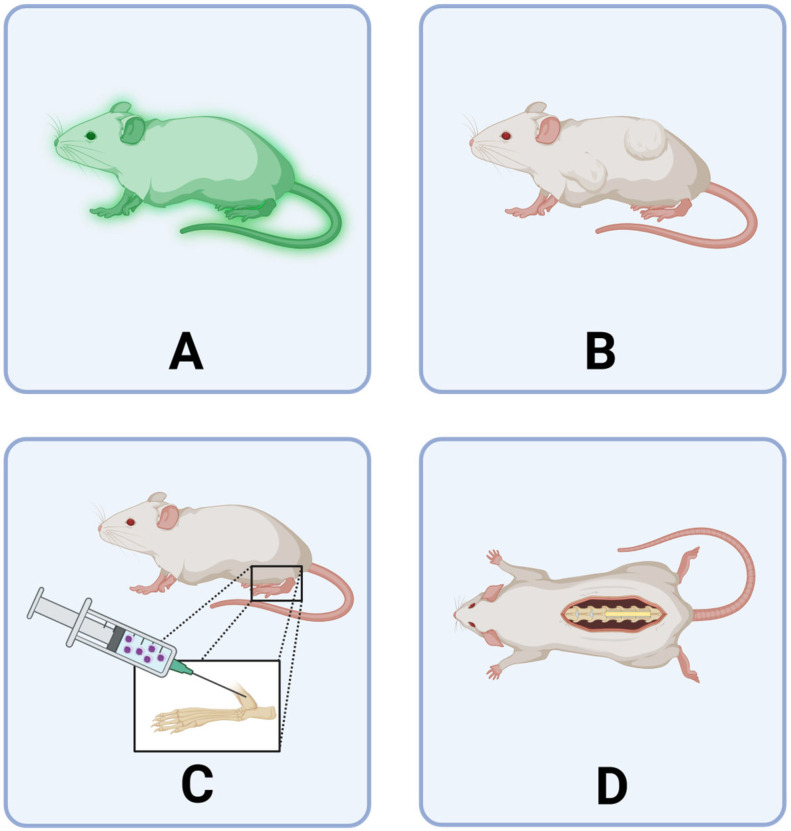
Rodent models used to study the bone marrow microenvironment for hematological malignancies. (**A**) Transgenic mice. Xenograft studies using human malignant cells implanted (**B**) subcutaneously or (**C**) orthotopically into immunocompromised mice. (**D**) Humanized 3D ossicle models. Generated using BioRender (https://BioRender.com/ah6a1v9 (accessed on 31 July 2025)).

Traditionally, the gold standard for modeling the bone marrow niche has been considered to be in vivo animal models. These models have been used to study the development and progression of a range of hematological malignancies, as well as to examine the effectiveness of treatments. A variety of transgenic and xenograft models, mostly in rodents, have been developed using different populations of human cells (Figure 2; Table 1). Such models have enabled the HSC–niche interactions to be examined both spatially and temporally through the use of sophisticated imaging technologies, including in vivo 2-photon microscopy and quantitative three-dimensional (3D) microscopy [31,32,33]. More recently, models incorporating humanized 3D ossicles, which involve scaffolds coated with human stromal cells implanted in immunodeficient mice, represent an improvement over more traditional transgenic or xenograft models as they attempt to bridge the gap between mouse and human [34,35,36,37]. These humanized models have overcome the incomplete cross-compatibility between murine and human stroma which has previously limited the rate of human HSC and hematological malignant cell engraftment. However, while these models have the benefit of being able to examine multiple cell types in relevant anatomical structures (including bones and the vasculature) concurrently, and recently developed humanized models have been shown to favor AML and HSC engraftment and mimic cellular distribution observed in human tissue, nevertheless, despite mimicking human diseased phenotypes, the underlying physiological and molecular mechanisms in these in vivo models may not be representative of the underlying pathology in humans [38], highlighting that improved models that better represent the human physiological condition are required. Despite these in vivo models being the ‘gold standard’, they still do not always translate into patient outcomes in terms of improved treatments, and views are emerging that alternative non-animal models may be more suitable, or at least complementary, for these types of investigations. While solid tumor in vivo models tend to be simpler to establish and have led to improved treatments, similar models for hematological malignancies are not as well established and physiologically translatable. Therefore, a variety of in vitro models have been developed, in an attempt to more closely mimic the human bone marrow microenvironment. Each of these models has various pros and cons, varies in complexity and clinical relevance, as well as suitability for various down-stream applications.

### 3.1. Two-Dimensional Co-Cultures

**Figure 3:**
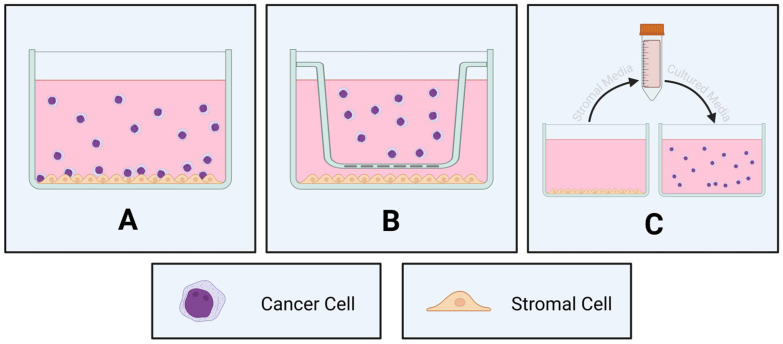
Two-dimensional co-culture models to examine the bone marrow microenvironment for hematological malignancies. (**A**) Direct co-culture—adherent stromal cells cultured in direct contact with cancer cells. (**B**) Indirect co-culture—adherent stromal cells separated from cancer cells through the use of an insert. (**C**) Conditioned medium—the medium is collected from wells containing stromal cells and used to culture cancer cells in separate wells. Generated using BioRender (https://BioRender.com/ny8ldfq (accessed on 31 July 2025)).

Tumor–stromal cell interactions play a significant role in cancer development and progression [44]. The majority of in vitro studies examining the role of the bone marrow microenvironment in cancer initiation, progression, and chemosensitivity have traditionally utilized conventional two-dimensional (2D) co-culture of cancer cells with progenitor cells on a supportive layer of bone marrow stromal cells. These co-culture models are broadly of two types: direct and indirect (Table 2). In direct co-culture, two subsets of cells—such as tumor cells and stromal, immune, or other supportive cells—are physically in contact with each other throughout the culturing process (Figure 3A). By contrast, in indirect co-culture, the two cell types are cultured in separate compartments (such as using an insert; Figure 3B), or conditioned medium of one cell type is used on the other cell type (Figure 3C). The direct co-culture model is more physiologically relevant, as it recapitulates the cell–cell contact that occurs within the natural biological environment. Although the indirect co-culture lacks cellular contact, this model still allows for the sharing of secreted factors between the different cell types. As such, these 2D co-culture models are useful for studying cell biology (such as cellular signaling, adhesion, and migration) within the bone marrow niche, and for examining the sensitivity of malignant cells to drugs in an in vitro model.

A variety of hematological malignancies and bone marrow components have been successfully integrated into 2D co-cultures to recapitulate aspects of the bone marrow niche that have proven particularly useful for studying malignant progression and drug sensitivity. For example, when primary bone marrow MSCs from chronic myeloid leukemia (CML) patients were co-cultured with CML patient blasts, enhanced CML blast survival was observed, as well as partial maintenance of leukemia stem cells (LSCs) [48], suggesting that this may be a useful model to study LSCs ex vivo. Additionally, to study the role of the immune system in leukemia progression and immunotherapy efficacy, AML cells have been co-cultured with macrophages and T cells ex vivo [43,51]. Using these novel co-culture models, it was shown that AML cells could be eliminated by activated macrophages in co-cultures, by inhibiting the ‘don’t eat me signal’ CD47 and increasing the ‘eat me signal’ CRT on the surface of AML cells to flag them for elimination by macrophages in vitro [51], which may help in characterizing new immunomodulatory therapies that may be useful for treating the disease.

The choice of bone marrow cells to be used for the co-culture is very important. For example, human primary MM cells grown in co-culture with bone marrow stromal cells isolated from MM patients secrete different factors compared to the same cells grown in co-culture with healthy bone marrow cells, the latter of which protected MM cells from dexamethasone, but not bortezomib, treatment [49]. While these patient-derived stromal cells offer physiological relevance, there are also limitations to their use, not least among them heterogeneity and the difficulty of obtaining sufficient material. As such, cultured human stromal cell lines, such as HS-5 and HS-27A, offer several advantages over primary bone marrow MSCs, including the provision of an abundant source of immortalized cells that can be manipulated to improve ease of downstream processing and analysis, all the while maintaining the ability to secrete important growth factors [53]. Additionally, a gene and pathway expression comparison with primary MSCs has demonstrated that HS-5 express MSC and immunomodulatory markers, except for CD106/VCAM-1, and that only the HS-5 cell line reproduces the MSC capacity to influence cancer biology and tumor immune escape mediated by stromal cells [54]. Indeed, 2D co-cultures of MM cells with HS-5 bone marrow stromal cells induced epigenetic and transcriptomic changes in the MM cells that can predict long-term outcomes, are associated with therapeutic resistance, and are reminiscent of extramedullary disease [50].

To incorporate some niche-related environmental factors, these cultures can also be grown under hypoxic conditions [55]. However, 2D models cannot precisely control oxygenation levels at the microscale. This has led to the development of microfluidic platforms to overcome this inherent limitation.

While these models have recapitulated part of the complex interactions between cancer and stromal cells, such as resistance to chemotherapy [56,57,58], and offer an improvement on traditional 2D cancer cell cultures, these co-culture systems have several limitations (Table 2), namely they do not incorporate structural elements of the bone marrow niche, including the extracellular matrix (ECM), molecular components, and growth factors, or take into account multiple bone marrow cell types. Additionally, other complex factors found in this niche, such as oxygen gradients [7], cannot be fully replicated in a 2D system. Furthermore, it has been long understood that proliferation rates and protein and gene expression levels do not match those of the original tumor [29,59]. Additionally, cellular components, particularly HSCs, do not behave the same in standard culture models as in the orthotopic environment. For example, HSCs rapidly differentiate when cultured under standard conditions [60], so modifications are required in these models, such as using scaffolds and soluble factors, to be able to accurately recapitulate these features. While the use of these soluble factors has helped to promote maintenance and expansion within this compartment, these systems often have limited success in growing HSCs with long-term engraftment capacity, so do not accurately reflect the in vivo microenvironment. Additionally, in a 2D co-culture model, the stromal cells are likely to be polarized as the basal surface of the cells will receive different signals from the apical side. As a result, more physiologically relevant 3D co-culture models have been developed and are beginning to be explored for a variety of applications.

### 3.2. Three-Dimensional Co-Cultures

It is vital that the tumor microenvironment is recapitulated in vitro to gain a better understanding of the role of the bone marrow niche in hematological cancer progression and sensitivity to chemotherapeutics. 3D co-cultures offer several advantages over conventional 2D cultures (Table 3). Importantly, cells grown in 3D culture produce alterations in cellular morphology, responses, metabolism, and hypoxic conditions that are reminiscent of the in vivo microenvironment in solid tumors [61,62].

**Figure 4:**
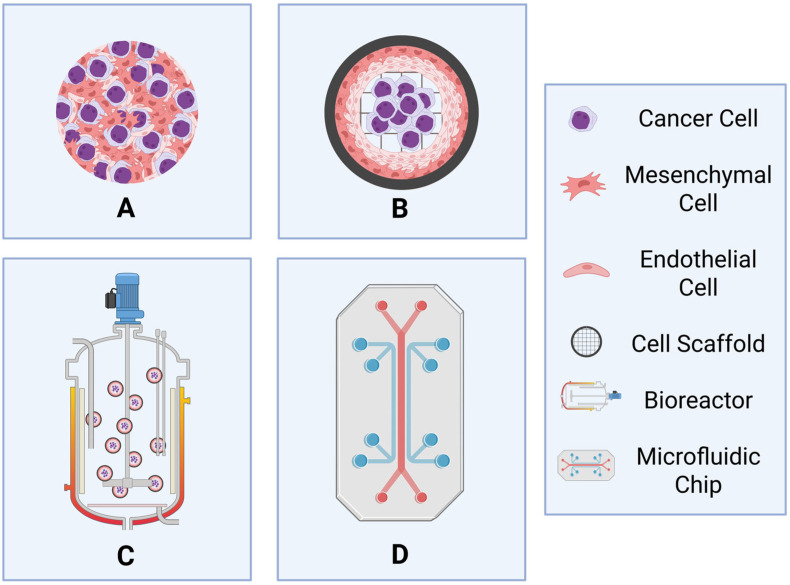
Examples of three-dimensional co-culture models to examine the bone marrow microenvironment for hematological malignancies. (**A**) Spheroid model—an anchorage-independent/scaffold-free static system composed of cancer, mesenchymal, and endothelial cells. (**B**) Organoid model—a scaffold-based static system composed of cancer, mesenchymal, and endothelial cells anchored to a cell mesh. (**C**) Bioreactor model—a dynamic model composed of cell microcarriers in constant motion to mimic blood flow. (**D**) Microfluidic model—a dynamic model that mimics bone marrow niches through the placement of cell types and the pumping of medium to replicate blood flow. Generated using BioRender (https://BioRender.com/manmheg (accessed on 31 July 2025)).

Although 3D co-cultures mimic aspects of in vivo tumor conditions, they still possess several limitations (Table 3). Firstly, they are more expensive and time-consuming than 2D co-cultures, factors that have limited their widespread application. Due to their more complex nature, downstream applications can prove tricky to examine, and they remain challenging to use for high-throughput preclinical investigation of drugs or for examining multiple drug combinations simultaneously. Additionally, scaffolds need to be carefully chosen to mimic aspects of the bone marrow niche (discussed further below). Despite these drawbacks, they are still useful models that more faithfully recapitulate the bone marrow microenvironment when compared to 2D co-cultures. 3D co-culture can involve static systems that examine anchorage-independent (scaffold-free) (Figure 4A) or anchorage-dependent (scaffold-based) growth of spheroids/organoids (Figure 4B), as well as dynamic systems, such as bioreactors and microfluidic systems, to mimic the dynamic microenvironment found in vivo (Figure 4C,D).

#### 3.2.1. Spheroids and Organoids

Spheroids are the simplest of 3D cultures, comprised of free-floating cell aggregates with little structural organization. Traditionally achieved by centrifugation or hanging drop method, the technique was first introduced in the 1970s [82] and became a popular way to mimic the nutrient and oxygen gradients experienced by cells of solid tumors. Spheroids can be formed from cell lines, primary cells, or tumor tissues, with or without the support of an ECM. Many leukemia cell lines spontaneously form into such clusters when grown in culture, and others, such as MM cells, can be induced in the presence of Matrigel [63]. Although clonal, these multicellular tumor spheroids allow for cell-to-cell contact and provide a clinically relevant model of chemotherapy resistance that can be used for large-scale drug screening. However, they can also be adapted into much more powerful tools through the integration of additional cell types, creating multicellular amalgamations that combine the cellular advantages of co-culturing with the additional benefit of biological interactions. Such a strategy was used to produce an adaptation of the widely used Eµ-TCL1 transgenic mouse model of CLL by using collagen to induce the formation of a stromal cell cluster by hanging drop, with later addition of murine Eµ-TCL1 leukemic cells [44]. This is particularly important for diseases like MM, where multiple cell types, such as adipose and osteoblasts contribute to the disease pathogenesis [83,84]. Further, a 3D tissue-engineered bone marrow co-culture derived from the bone marrow supernatant of MM patients has been shown to better simulate the interaction between MM cells and the microenvironment, and also to induce higher levels of chemoresistance [67].

Compared to spheroids, organoids are much more complex structures containing different cell lineages that more closely reflect the structure and function of an organ. They are most commonly formed from stem cells but may also be induced to form ex vivo from tumor cells or tissues and generally require ECM and growth factors to stimulate differentiation. While blood cells themselves don’t form specialized structures, previously established organoid models can nevertheless be used to recapitulate their microenvironment. This has been exemplified in human MM cells cultured with or without the addition of ex vivo derived MSCs and endothelial progenitor cells (EPCs) in a 3D bone marrow niche model. When cultured in Matrigel, the MSC and EPCs formed a 3D matrix of tubules resembling a perivascular network, which led to increased MM cell line proliferation and viability, and has been shown to be suitable for the examination of novel drug delivery systems [66,85]. Importantly, induced pluripotent stem-cell-derived bone marrow organoids (iPSC-BMO) support the implantation and survival of a range of malignant hematological cells, including MM, ALL, CML, AML or MDS cells from patients, and when xenotransplanted in mice demonstrate transient engraftment potential [64,65,86].

Similarly, organoids have successfully been used to study microenvironment signaling pathways involved in the progression of diffuse large B-cell lymphoma (DLBCL), B cell responses in patients with lymphoma, drug screening in non-Hodgkin’s lymphoma, interaction of DLBCL cells with the microenvironment, and also for examining T-cell-mediated immune responses and to examine novel immunotherapies in follicular lymphoma [68,69,70,71,72,73,74].

While these organoid co-culture models have been recently developed for MM, MDS and lymphomas, their use in leukemias is much less widespread. Two-dimensional co-culture or in vivo models have long been the standard for examining leukemia cell interactions with the bone marrow niche; however, recent 3D co-culture systems have been developed for T-cell acute lymphoblastic leukemia (T-ALL) and B-cell acute lymphoblastic leukemia (B-ALL), whereby patient samples have been co-cultured with stromal or osteoblast cells to generate 3D organoids in vitro [75,76,87]. These models provide a valuable platform for examining the pathogenesis of ALL relapse, as well as drug sensitivity screenings. Bone-marrow-derived MSCs co-cultured with AML cells in 3D systems exhibited higher resistance to chemotherapy than for the 2D co-culture model [88,89], demonstrating the importance of the architecture in determining treatment response. Further, a 3D triculture (endothelial and mesenchymal stromal cells with AML cells) further increased chemoresistance when compared to 3D and 2D bi- and monocultures [90]. By contrast, AML cells cultured with healthy osteogenic cells are sensitive to chemotherapeutics [91]. Taken together, these studies suggest that in leukemias, the cell types used for co-culture have a more pronounced impact on chemosensitivity than just a switch in dimensionality from 2D to 3D.

While these organoid co-culture models represent a significant improvement in in vitro modeling, they still possess limitations compared to traditional 2D co-culture methods. Despite the benefits of incorporating multiple cell types, paracrine and autocrine signals, and allowing the examination of cell–cell and cell–ECM contacts, 3D models still lack some of the key components of the in vivo bone marrow niche, including a failure to mimic the nutrient/waste gradients and the dynamic environments that are observed in vivo. From an experimental viewpoint, 3D organoids are complex structures, requiring more time to establish, validate, and recreate, while downstream applications require more optimization than for 2D co-cultures. Extracting cells from the 3D co-culture is difficult, and can result in altered cellular morphology and characteristics, ultimately defeating the original purpose of the model. Despite the complexities these 3D models present, they appear to be more physiologically relevant than traditional 2D co-cultures and therefore may be useful in bridging the gap between preclinical examination of new drugs, and clinical investigation.

#### 3.2.2. Importance of Scaffold Choice

In terms of scaffold-based 3D co-culture systems, the most common scaffolds can be further characterized as either biological or synthetic. Biological scaffolds, including collagens, Matrigel, and decellularized tissues, contain ECM components and therefore resemble the tumor microenvironment. However, these materials are highly heterogeneous between different manufacturing lots or tissues of origin and can impede reproducibility. By contrast, synthetic scaffolds, including gelatin, cellulose, alginate, bioceramics, and hydrogels are comprised of biologically compatible polymers and hydrogels. While they are not as biologically faithful as biological scaffolds, they have the benefit of being highly uniform and reproducible. An ideal scaffold needs to provide an appropriate environment for cell adhesion, proliferation, migration and differentiation to most accurately recapitulate the bone marrow ECM. The scaffolds need to be designed with properties similar to those of the bone marrow niche, including surface-area-to-volume ratio, adhesive features, and pore size. Hydrogel-based scaffolds are often used, as their mechanical properties, which can have a significant effect on cells, such as controlling MSC differentiation [92], can be tailored to replicate the ECM [93].

Different regions of the bone marrow niche are comprised of different proteins. The endosteal niche contains type I and IV collagen, fibronectin and osteopontin, the central niche contains laminin, heparin, fibronectin, and hyaluronic acid, whereas the perivascular region contains type IV collagen, laminin and fibronectin [79]. Therefore, to accurately mimic each of these different niches; different scaffolding materials are required. Another consideration is that some of these materials have been shown to be inadequate for hematopoietic progenitor cell growth or function. For example, even though polylactic-co-glycolic acid (PLGA) is a biodegradable and biocompatible material, it does not support the growth of CD34+ cells [94], making it inappropriate to use in these models. By contrast, the biodegradable zwitterionic hydrogel, the synthetic polycaprolactone, polyurethane, polyethylene glycol biofunctionalized hydrogels, bio-derived bone scaffolds, gelatin-based porous scaffold functionalized with stromal cells, non-woven polyester fiber/polypropylene mesh, as well as the natural materials collagen, fibrin and cellulose all support CD34+ cell function [36,94,95,96,97,98,99,100], and ceramic scaffolds biofunctionalized with mesenchymal cells and osteoblasts produced a bone marrow-like environment where HSCs were capable of hematopoietic reconstitution [101], all of which are therefore highly suitable for use in these models.

Despite their wide use in multiple in vitro models, as well as their suitability for culturing particular bone marrow cell types, these scaffolds lack the complexity of the native ECM, and present challenges in relation to biocompatibility. By contrast, natural scaffolds obtained from decellularized tissue maintain the architecture of the native tissue, are biocompatible and allow for the simulation of the niche architecture that is as close to the natural environment as possible. For example, decellularized ECM and bovine or porcine bone marrow enhance HSC and MSC adhesion, proliferation and differentiation, decellularized Wharton’s jelly matrix decreases proliferation and differentiation of leukemia cells and induces chemoresistance, and ossified 3D bone tissue supports the ex vivo expansion of MM cells [102,103,104,105,106]. However, decellularization can be achieved via multiple methods, and the method of treatment can impact downstream applications as well as the biomechanical characteristics of the remaining scaffold, so the choice of technique is a very important consideration.

#### 3.2.3. Dynamic 3D Models

Although the 3D models discussed above come closer to recreating the physical structure of the bone marrow microenvironment, they remain static and lack the dynamic mechanical stimulation experienced by cells within a living body. Dynamic cell culture models attempt to recreate the natural blood flow and continual shear stress experienced by living cells in vivo. Bioreactors achieve this through constant rotation of the growth medium, either horizontally in a spinner flask equipped with a stirring rod, or vertically in a rolling wall bioreactor. Such a strategy is illustrated by a CLL patient cell 3D co-culture model based on the integrated use of HS-5 stromal cell-repopulated Spongostan scaffolds and a rotating bioreactor [77], where primary CLL cells were recovered from both inside and outside the scaffolds and reliably reproduced in vivo homing and migration events. This model could be used to allow a better understanding of the mechanisms underpinning CLL dissemination and homing to the bone marrow and potentially elucidate the pathogenic mechanisms underpinning CLL relapse and response to treatment. As with the static 3D culture systems described above, multiple cell types can be incorporated in the microcarrier scaffolds to create the most relevant culture model [37]. Alternatively, bioreactors have been successfully used to culture naturally heterogeneous tissue explants from both normal and pathological bone marrow [78].

Microfluidic cell culture systems represent the most advanced and physiologically relevant models to date, incorporating a pumping system to continuously deliver cell culture media through a miniature cell-growing carrier at a controlled rate of shear stress. By combining multiple cell types, the concept of ‘organs on chips’ or ‘tissue chips’ was developed. Leukemia-on-a-chip models are most commonly created using the soft lithography method, with a polydimethylsiloxane (PDMS) material [79]. Leukemia-on-a-chip models drive differentiation of progenitor cells further than static 3D models [80]. Additionally, these dynamic models closely mimic the in vivo microenvironment and may be useful in reducing/eliminating the use of in vivo animal models to study the role of the bone marrow niche in hematological malignancies, particularly in relation to disease pathogenesis and progression. An in vitro organotypic ‘leukemia-on-a-chip’ model of B-ALL that mimics the periosteal, perivascular and central sinus bone marrow zones has been generated using human osteoblasts, MSCs, and endothelial (HUVEC) cells [81]. This model has been utilized to demonstrate the niche-specific chemoresistance mechanisms for B-ALL and will be a useful screening tool for personalized medicine and treatment response prediction.

While these dynamic models, particularly leukemia-on-a-chip models, offer a physiological improvement over static models, due to the need for specialist equipment, they can be expensive to establish, and their dynamic nature means that the architecture can be disrupted. While they are useful and relevant model systems for studying disease pathogenesis and progression, due to their nature, they are not suitable for high-throughput examination of novel drugs, though they may be useful for treatment response prediction.

## 4. Important Considerations and Future Directions

Preclinical models that accurately mimic the bone marrow microenvironment in hematological malignancies are important tools in elucidating the pathophysiology of these malignancies, as well as investigating the efficacy of novel therapies. Recent years have seen improvements in the cellular and molecular complexity of these models; however, the ability to study multiple cell types simultaneously, the movement of cells, and the impact of anatomical structures still require more development, particularly for the examination of multiple drug combinations, so that optimal doses can be identified. Additionally, improvement in these models will lead to an increased understanding of the pathogenic mechanisms leading to the homing, dissemination, and relapse of malignant cells, potentially leading to the identification of novel therapeutic targets for the treatment of relapsed disease.

Further, as the bone marrow niche contains several distinct compartments: including the osteoblastic niche that supports HSC quiescence and self-renewal, and the vascular/perisinusoidal niche that promotes HSC proliferation and differentiation, the most relevant niche for the hematological malignancy being investigated needs to be utilized. Each of these niches comes with distinct cellular, physical, and chemical requirements, which need to be considered when considering which model would be most suitable for the question being asked.

Models that combine multiple bone marrow cell types as well as malignant cells, in a 3D dynamic system, will provide the most accurate representation of the bone marrow niche. Choosing the most relevant bone marrow cell types will be dependent on the cancer type being examined, and the choice of scaffolding is an important consideration that must be made to ensure that these cell types are adequately supported, and the niche of interest is modeled correctly. Each of the existing preclinical co-culture models has advantages as well as limitations, and the best model to answer research questions will vary with cancer type as well as the downstream applications that will be examined. The main limiting factors when considering which model to use include access to appropriate equipment and resources, as well as suitability of the model for the desired downstream application. For example, physiologically relevant models of the bone marrow niche hematological cell microenvironment suitable for high-throughput screening of multiple drug combinations simultaneously are still lacking and require further development to be able to bridge the preclinical-to-clinical gap.

These models offer potential strategies for personalized medicine, where an ex vivo platform that simulates the bone marrow niche microenvironment of malignant cells can be used to examine treatment efficacy to a variety of anti-cancer agents. In the clinical setting, such a platform should be easy to perform, reproducible, and able to simultaneously process many samples.

## 5. Conclusions

Developing ex vivo models that closely replicate the in vivo bone marrow niche are urgently required. Three-dimensional co-culture models with multiple bone marrow niche cell types and malignant hematological cells are a useful tool to bridge the gap between in vitro and in vivo studies. These models will also be relevant for screening sensitivity to new therapies and perhaps predicting patient response to these therapies; however, adaptations to screen multiple combinations simultaneously will need to be developed to be most beneficial clinically. These 3D co-culture models could be used as the initial template for screening drug sensitivity prior to moving into in vivo models or clinical studies.

## Figures and Tables

**Table 1:** Summary of preclinical animal models of the bone marrow microenvironment, including their suitable applications in hematological cancer research.

Summary of Model	Advantages	Limitations	Uses	References
Genetically modified	Physiologically relevant; same species; can examine the role of the immune system and native components in hematological malignancies	Expensive; not necessarily relevant to humans; not suitable for high-throughput drug screening	Pathogenesis; drug sensitivity	[32,39,40,41,42]
Xenograft/implantation	May be physiologically relevant (if orthotopic or involving humanized ossicles); can examine role of multiple cell types simultaneously in physiologically relevant situations	Expensive; not necessarily equivalent to humans; cannot examine the role of the immune system; not suitable for high-throughput drug screening	Drug sensitivity; progression	[31,34,35,36,37,43]

**Table 2:** Summary of preclinical two-dimensional (2D) in vitro models of the bone marrow microenvironment, including their suitable applications in hematological cancer research.

Summary of Model	Advantages	Limitations	Uses	References
**Direct co-culture:** cancer cells are grown directly on bone marrow stromal cell monolayers	Easy and inexpensive to establish; can study the effect of stromal cells on cancer cells and vice versa; can examine the impact of cell–cell contact; more relevant than 2D single-cell culture; allows the study of homogeneous populations	Does not contain multiple cell types; lacks 3D or anatomical factors associated with the niche; unable to perform downstream assays separately for each cell type; not possible to perform high-throughput assays for drug treatments	Examines cell biology and signaling processes involved in relapse and drug sensitivity (adhesion, migration, proliferation); allows the examination of drug sensitivity	[43,45,46,47,48,49,50]
**Indirect co-culture:** cancer cells are grown indirectly with microenvironment cells separated by a permeable membrane or insert	Easy and inexpensive to establish; can study the effect of microenvironment cells on cancer cells and vice versa; allows the sharing of secreted factors through a permeable membrane; allows the study of homogeneous populations	Lacks cell–cell contact; does not contain multiple cell types; lacks 3D or anatomical factors associated with the niche; not possible to perform high-throughput assays for drug treatments	Examines signaling processes involved in bone marrow microenvironment; allows the examination of drug sensitivity	[51,52]
**Indirect co-culture (media):** cancer or microenvironment cells are grown in conditioned media from another cell type	Easy and inexpensive to establish; can study the effect of secreted factors on different cell types; allows the study of homogeneous populations	Lacks cell–cell contact; does not contain multiple cell types; lacks 3D or anatomical factors associated with the niche	Allows the examination of drug sensitivity; examines the effect of secreted biomolecules on other cell types	[47]

**Table 3:** Summary of preclinical three-dimensional (3D) in vitro models of the bone marrow microenvironment, including their suitable applications in hematological cancer research.

Model	Summary of Model	Advantages	Limitations	Uses	References
Static 3D co-culture	**Scaffold-free:** cancer and microenvironment cells are grown without a scaffold and are allowed to morph into spheroids/organoids in the absence of an anchor	More physiologically relevant than the 2D model; does not require specialized equipment; can study cell–cell interactions	Does not contain multiple cell types; lacks ECM–cancer cell interactions; time-consuming; grown under static conditions; not suitable for high-throughput drug screening	Examines signaling processes; drug sensitivity	[44,61,62,63,64,65,66,67,68,69,70,71,72,73,74,75,76]
	**Scaffold-based:** cancer and microenvironment cells are grown in the presence of a synthetic or biological scaffold and are allowed to grow as spheroids/organoids	More accurately recapitulates the bone marrow microenvironment; can study cell–cell and cell–ECM interactions	Time-consuming; expensive; grown under static conditions; not suitable for high-throughput drug screening	Examines signaling processes; drug sensitivity	
Dynamic 3D co-culture	**Bioreactor:** uses a 3D bioreactor to grow cancer cells, microenvironment cells, and scaffolds	Can examine multiple cell types; can study cell–cell and cell–ECM interactions; grown under dynamic conditions	Expensive and requires specialized equipment; time-consuming; dynamic growth conditions can disrupt cells or the scaffold architecture; not suitable for high-throughput drug screening	Examines signaling processes; drug sensitivity	[77,78]
	**Microfluidics:** uses a 3D bioreactor to grow cancer cells, microenvironment cells, and scaffolds (mimics osteoblastic and vascular niches)	Can examine multiple cell types; can examine multiple niches simultaneously; can study cell–cell and cell–ECM interactions; grown under dynamic conditions	Expensive and requires specialized equipment; time-consuming; dynamic growth conditions can disrupt cells or the scaffold architecture; not suitable for high-throughput drug screening	Model processes involved in progression and relapse; drug sensitivity	[79,80,81]

ECM: Extracellular matrix.

## Data Availability

No new data were created.

## Abbreviations

The following abbreviations are used in this manuscript:

2D	Two-Dimensional
3D	Three-Dimensional
ALL	Acute Lymphoblastic Leukemia
AML	Acute Myeloid Leukemia
B-ALL	B-cell Acute Lymphoblastic Leukemia
CLL	Chronic Lymphocytic Leukemia
CML	Chronic Myeloid Leukemia
DLBCL	Diffuse Large B-cell Lymphoma
ECM	Extracellular Matrix
HSC	Hematopoietic Stem Cell
iPSC-BMO	Induced Pluripotent Stem-Cell-Derived Bone Marrow Organoids
LSC	Leukemia Stem Cell
MDS	Myelodysplastic Syndromes
MM	Multiple Myeloma
MPN	Myeloproliferative Neoplasms
MSC	Mesenchymal Stem Cell
PDMS	Polydimethylsiloxane
T-ALL	T-cell Acute Lymphoblastic Leukemia
